# Quantifying Hemodynamic Cardiac Stress and Cardiomyocyte Injury in Normotensive and Hypertensive Acute Heart Failure

**DOI:** 10.3390/biomedicines12051099

**Published:** 2024-05-16

**Authors:** Nikola Kozhuharov, Eleni Michou, Desiree Wussler, Maria Belkin, Corinna Heinisch, Johan Lassus, Krista Siirilä-Waris, Harjola Veli-Pekka, Nisha Arenja, Thenral Socrates, Albina Nowak, Samyut Shrestha, Julie Valerie Willi, Ivo Strebel, Danielle M. Gualandro, Katharina Rentsch, Micha T. Maeder, Thomas Münzel, Mucio Tavares de Oliveira Junior, Arnold von Eckardstein, Tobias Breidthardt, Christian Mueller

**Affiliations:** 1 Department of Cardiology and Cardiovascular Research Institute Basel (CRIB), University Hospital Basel, University of Basel, Petersgraben 4, 4031 Basel, Switzerland; 2 Department of Cardiology, University Hospital Bern, University of Bern, Freiburgstrasse 20, 3010 Bern, Switzerland; 3 Department of Internal Medicine, University Hospital Basel, University of Basel, 4001 Basel, Switzerland; 4 Heart and Lung Center, Department of Cardiology, Helsinki University Central Hospital, 00280 Helsinki, Finland; 5 Department of Emergency Care, Helsinki University Hospital, 00280 Helsinki, Finland; 6 Department of Cardiology, Solothurner Spitäler AG, 4500 Solothurn, Switzerland; 7 Department of Endocrinology and Clinical Nutrition, University Hospital Zurich, 8091 Zürich, Switzerland; 8 Heart Institute (INCOR), University of Sao Paulo Medical School, Sao Paulo 01246-000, Brazil; 9 Department of Laboratory Medicine, University Hospital Basel, 4031 Basel, Switzerland; 10 Department of Laboratory Medicine, University Hospital Zurich, 8091 Zürich, Switzerland; 11 Department of Cardiology, Kantonsspital St. Gallen, 9000 St. Gallen, Switzerland; 12 University Medical Center, Johannes Gutenberg University Mainz, 55122 Mainz, Germany; 13 Institute for Emergency Medicine, University Hospital Zurich, University of Zurich, 8006 Zürich, Switzerland

**Keywords:** acute heart failure, pathophysiology, natriuretic peptides, cardiac troponin

## Abstract

Background: The characterization of the different pathophysiological mechanisms involved in normotensive versus hypertensive acute heart failure (AHF) might help to develop individualized treatments. Methods: The extent of hemodynamic cardiac stress and cardiomyocyte injury was quantified by measuring the B-type natriuretic peptide (BNP), N-terminal proBNP (NT-proBNP), and high-sensitivity cardiac troponin T (hs-cTnT) concentrations in 1152 patients presenting with centrally adjudicated AHF to the emergency department (ED) (derivation cohort). AHF was classified as normotensive with a systolic blood pressure (SBP) of 90–140 mmHg and hypertensive with SBP > 140 mmHg at presentation to the ED. Findings were externally validated in an independent AHF cohort (n = 324). Results: In the derivation cohort, with a median age of 79 years, 43% being women, 667 (58%) patients had normotensive and 485 (42%) patients hypertensive AHF. Hemodynamic cardiac stress, as quantified by the BNP and NT-proBNP, was significantly higher in normotensive as compared to hypertensive AHF [1105 (611–1956) versus 827 (448–1419) pg/mL, and 5890 (2959–12,162) versus 4068 (1986–8118) pg/mL, both *p* < 0.001, respectively]. Similarly, the extent of cardiomyocyte injury, as quantified by hs-cTnT, was significantly higher in normotensive AHF as compared to hypertensive AHF [41 (24–71) versus 33 (19–59) ng/L, *p* < 0.001]. A total of 313 (28%) patients died during 360 days of follow-up. All-cause mortality was higher in patients with normotensive AHF vs. patients with hypertensive AHF (hazard ratio 1.66, 95%CI 1.31–2.10; *p* < 0.001). Normotensive patients with a high BNP, NT-proBNP, or hs-cTnT had the highest mortality. The findings were confirmed in the validation cohort. Conclusion: Biomarker profiling revealed a higher extent of hemodynamic stress and cardiomyocyte injury in patients with normotensive versus hypertensive AHF.

## 1. Introduction

Acute heart failure (AHF) is the most common diagnosis in the emergency department (ED) leading to hospitalization [1,2]. In contrast to chronic heart failure, where relevant improvements in therapy and prognosis have been achieved, morbidity and mortality in AHF remain unacceptably high [1,2]. This may be at least partly due to an incomplete understanding of the pathophysiological mechanisms involved in the different AHF syndromes [3,4,5]. Attempts were undertaken to develop an AHF classification, taking into account that AHF is not a uniform disease but a heterogeneous syndrome [2].

Among the proposed classifications of AHF, the most attractive are those based on a clinical assessment at presentation, allowing for a more precise risk stratification and helping to guide “individualized” therapeutic strategies. According to the systolic blood pressure (SBP) at presentation, there is a differentiation between hypertensive (>140 mmHg), normotensive (90–140 mmHg), and hypotensive (<90 mmHg) AHF [2,3,6]. The latter group represents only 5–8% of AHF patients and is associated with a dismal prognosis [2,7,8]. Unfortunately, the characteristics of the two other entities and their underlying pathophysiology are only partly understood [6]. In this respect, two mechanisms seem to play a major role: hemodynamic cardiac stress and cardiomyocyte injury [9]. Cardiovascular biomarkers incorporated in clinical practice enable to accurately quantify these mechanisms: First, plasma concentrations of the B-type natriuretic peptide (BNP) and N-terminal pro-B-type natriuretic peptide (NT-proBNP) reflect the severity of the underlying hemodynamic cardiac stress [10,11]. Second, plasma concentrations of high-sensitivity cardiac troponin (hs-cTn) allow for the quantification of cardiomyocyte injury [12,13].

We, therefore, aimed to characterize the distinct clinical phenotypes of normotensive and hypertensive AHF by quantifying hemodynamic cardiac stress and cardiomyocyte injury using biomarkers as non-invasive quantitative tools.

## 2. Materials and Methods

### 2.1. Study Population and Design

Basics in Acute Shortness of Breath EvaLuation (BASEL V) (ClinicalTrials.gov registry, number NCT01831115) was a prospective, multicenter, diagnostic study enrolling adult patients presenting with acute dyspnea of not traumatic cause to the ED of two university hospitals (Basel and Zurich) in Switzerland [14]. In addition to consecutive patients enrolled in BASEL V, for this prognostic analysis, patients from tertiary hospitals in Basel, Lucerne, St. Gallen (Switzerland), Mainz (Germany), and Sao Paolo (Brazil) with an adjudicated final diagnosis of AHF enrolled in an AHF therapy study were also eligible if blood studies were available for the biomarker measurements prior to the study’s treatment initiation (ClinicalTrials.gov registry, number NCT00512759) [15]. Patients eligible for this analysis were enrolled between November 2006 and August 2015, when heart failure treatment with sacubitril/valsartan and sodium glucose co-transporter 2 inhibitors (SGLT2 inhibitors) was not yet implemented in clinical practice. First, systolic blood pressure measured at presentation to the ED was used to allocate patients to the hypertensive AHF group. According to the systolic blood pressure (SBP) at presentation, patients were allocated to the hypertensive (>140 mmHg) or normotensive (90–140 mmHg) AHF groups. Patients with hypotensive (<90 mmHg) AHF were not considered for this analysis, as they represented a phenotype with a particularly dismal prognosis. Patients without available data on blood pressure measurements at admission were excluded from this analysis. While enrolment was independent of renal function, patients with end-stage renal disease on chronic dialysis were excluded.

The study was carried out according to the principles of the Declaration of Helsinki and was approved by the local ethics committees. Written informed consent was obtained from all participants. The authors designed the study, gathered, and analyzed the data, vouch for the data and analysis, wrote the paper, and decided to publish.

### 2.2. Adjudication of the AHF Diagnosis

The final discharge diagnosis was adjudicated by two independent cardiologists/internists using all the available medical records pertaining to the patient (including clinical history, physical examination, 12-lead ECG; laboratory findings, including BNP or NT-proBNP; chest X-ray; echocardiography findings, including systolic and diastolic function [16]; lung function testing, computed tomography, the response to therapy, and autopsy data in those patients who died in-hospital) from ED presentation until the follow-up period of up to 360 days. Regarding laboratory findings, all parameters obtained through the clinician’s routine diagnostic workup were taken into account. These included one of the natriuretic peptides (BNP or NT-proBNP) with a class I recommendation in current guidelines [2]. The diagnostic criteria suggested by the relevant European practice guidelines were applied. In situations of disagreement about the final diagnosis, cases were reviewed and adjudicated in conjunction with a third cardiologist/internist.

### 2.3. Initial Clinical Evaluation and Follow-Up

Patients underwent an initial clinical assessment at ED, including a clinical history, physical examination, ECG, pulse oximetry, blood tests, and chest X-ray. Echocardiography during the index hospitalization, coronary angiography, and a cardiac MRI were performed at the discretion of the attending physician. Measurements of left ventricular internal diameters at end-diastole and end-systole were obtained from the parasternal long-axis view, and the LVEF was calculated using the biplane method of disks (modified Simpson’s rule).

Patients were contacted after 90 days and at 1 year after discharge by telephone or in written form by trained researchers who were unaware of the patients’ blood pressure at presentation in the ED during the index hospitalization. In case of a possible relevant medical event, such as death or HF rehospitalization, further information was obtained from the hospital medical records, the general practitioner of the patient, or the national death registry.

### 2.4. Biochemical Measurements

At the patient’s presentation to the ED, blood samples were collected in tubes containing heparin or potassium ethylenediaminetetraacetic acid (EDTA) according to the biomarker determination. These study blood samples were frozen at −80 °C until being assayed in a dedicated core laboratory.

Plasma NT-proBNP (or BNP) concentrations were available to the adjudicating physicians. NT-proBNP plasma concentrations were measured using the Elecsys proBNP assay (Elecsys proBNP, Roche Diagnostics AG, Rotkreuz, Switzerland). The intra-assay CV ranged from 1.8% to 2.7% and from 2.4% to 3.2% for within-run and total imprecision, respectively [17]. Plasma BNP concentrations were measured using the Axsym or Architect BNP assays (Abbott Laboratories, Baar, Switzerland).

High-sensitivity cardiac troponin T (hs-cTnT) was measured using the Elecsys troponin T assay (Elecsys 2010, Roche Diagnostics, Rotkreuz, Switzerland), which has a 99th percentile concentration of 14 ng/L with a corresponding CV of 10% at 13 ng/L. Limit of blank and limit of detection were determined to be 3 ng/L and 5 ng/L [18], respectively.

### 2.5. Validation Cohort

Consecutive patients presenting with AHF at 14 university, central, and regional hospitals in Finland were enrolled between February and May 2004 into the Finnish Acute Heart Failure Study (FINN-AKVA), which served as the validation cohort. Clinical characteristics of the study population were presented previously [19,20]. The diagnosis of AHF was confirmed at discharge before inclusion in the cohort. Long-term mortality rates were assessed using the Finnish National Population Register. Patients with NT-proBNP and cardiac troponin measurements were considered eligible for this analysis. Biomarkers were measured using the Roche Diagnostics Elecsys NT-proBNP assay, the Roche Diagnostics Elecsys^®^ 2010 fourth generation cTnT assay, and the Abbott Architect^®^ cardiac troponin I (cTnI) assay [12,19,21].

### 2.6. Statistical Methods

The Kolmogorov–Smirnov test was used for testing normality. Since the vast majority of continuous variables did not have a normal distribution, they were presented as medians with an interquartile range (IQR), and categorical variables as numbers and percentages. Comparisons between groups were determined using the Chi-square test, Mann–Whitney U test, and Kruskal–Wallis test, as appropriate. Spearman’s rho was used to analyze correlations. Survival during follow-up was plotted in Kaplan–Meier curves, and the log-rank test was used to assess differences between groups. Normotensive AHF and further pre-defined variables from a validated risk model to predict all-cause mortality were entered in the multivariable Cox proportional hazard model [22]. In contrast to the validated risk model for the prediction of the combination of all-cause mortality, high-density lipoprotein plasma concentrations at baseline were not available. Furthermore, all baseline characteristics from Table 1 were tested in univariable models to predict all-cause mortality. Variables that were significant predictors in the univariable models were then entered into a multivariable model. In the absence of an established prognostic cut-off concentration [2], the median NT-proBNP as well as BNP cut-off concentration in the derivation cohort were used to differentiate between patients with a high vs. low natriuretic peptide concentration at presentation. For hs-cTnT, 14 ng/L (the upper limit of normal) and the median in the derivation cohort were used as cut-offs [18]. All hypothesis testing was conducted 2-sided and a *p*-value < 0.05 was regarded as significant. Statistical analyses were performed by using SPSS/PC software package (version 25.0).

## 3. Results

### 3.1. Patients’ Demographics and Characteristics

A total of 1152 patients from the derivation cohort were eligible for this analysis (Supplemental Figure S1). The clinical characteristics of the derivation cohort are presented in Table 1. The median age was 79 years and 43% of patients were women, with less women in the hypertensive AHF group. Roughly half of the patients had a history of heart failure and 82% had known hypertension. The body mass index and body surface area did not significantly differ in both groups.

Normotensive AHF was present in 667 patients (58%). The number of de novo heart failure presentations was significantly lower in the normotensive AHF group than in the hypertensive AHF group, and a clinical history of hypertension was less likely. Heart failure with reduced left ventricular ejection fraction (LVEF < 40%, HFrEF) was more common and left ventricular end-diastolic diameter was significantly higher among normotensive AHF patients. Furthermore, among those with normotensive AHF, more patients reported weight gain and a longer duration of worsening shortness of breath. The creatinine plasma concentration was also higher in the normotensive AHF group.

### 3.2. Hemodynamic Cardiac Stress and Cardiomyocyte Injury

There was a significant difference in hemodynamic cardiac stress, as quantified by the BNP as well as NT-proBNP plasma concentrations, between normotensive and hypertensive AHF patients with higher concentrations in the normotensive group (BNP: 1105 pg/mL versus 827 pg/mL, *p* < 0.001; NT-proBNP: 5890 versus 4068 pg/mL, *p* < 0.001; Table 2). There was a weak inverse correlation between SBP and NT-proBNP at presentation (r = −0.152, *p* < 0.001). The correlation between SBP and BNP concentration was also weak (r = −0.166, *p* < 0.001). There was no significant correlation between SBP and hs-cTnT (r = −0.014, *p* = 0.664). The change in BNP and NT-proBNP plasma concentrations through the course of hospitalization did not differ significantly between normotensive and hypertensive AHF patients (*p* = 0.609 and *p* = 0.101, respectively). Notably, there was a significant difference in the change in hs-cTnT throughout the course of hospitalization between the normotensive and hypertensive AHF groups (1 (0.9–13) vs. 5 (4–14) pg/mL, *p* = 0.039) with a more pronounced decrease in hypertensive than normotensive AHF.

In a multivariable logistic regression analysis including the variables normotensive AHF, age, sex, history of heart failure, history of hypertension, creatinine plasma concentration, and LVEF, normotensive AHF was not an independent predictor of a BNP or NT-proBNP concentration above the median (Table S1A,B).

As quantified using the hs-cTnT assay, cardiomyocyte injury was higher in normotensive AHF patients than hypertensive AHF patients (41 versus 33 ng/L, *p* < 0.001, Table 2). In a multivariable regression model including the variables normotensive AHF, age, sex, history of heart failure, creatinine plasma concentration, and LVEF, normotensive AHF was not an independent risk factor for elevated hs-cTnT (Table S2A,B).

In the overall population, there was a weak correlation between the extent of hemodynamic cardiac stress, as quantified by a natriuretic peptide, and the extent of cardiomyocyte injury, as quantified by hs-cTnT, (BNP: r = 0.074, *p* = 0.012; NT-proBNP: r = 0.146, *p* < 0.001). The correlations were comparable in patients with normotensive AHF (BNP: r = 0.076, *p* = 0.051, NT-proBNP: r = 0.115, *p* = 0.004) and those with hypertensive AHF (BNP: r = 0.094, *p* = 0.038, NT-proBNP: r = 0.198, *p* < 0.001).

### 3.3. Heart Failure Therapy in Relation to Hemodynamic Cardiac Stress and Cardiomyocyte Injury Patterns

Patients with a higher extent of hemodynamic cardiac stress or cardiomyocyte injury were more often normotensive (Table S3, *p* < 0.001 for both). Medical therapy on admission and at discharge is presented in Table S4. At presentation, significantly more patients with normotensive AHF were on chronic heart failure medication compared to those with hypertensive AHF. During hospitalization, guideline-directed medical therapy was initiated more often in hypertensive AHF; at discharge, differences in the proportions of patients receiving renin–angiotensin system blockers, beta-blockers, and aldosterone antagonists were no more present. Notably, no patients were treated with sacubitril/valsartan or SGLT2 inhibitors.

### 3.4. Mortality in Relation to Blood Pressure

**Figure 1:**
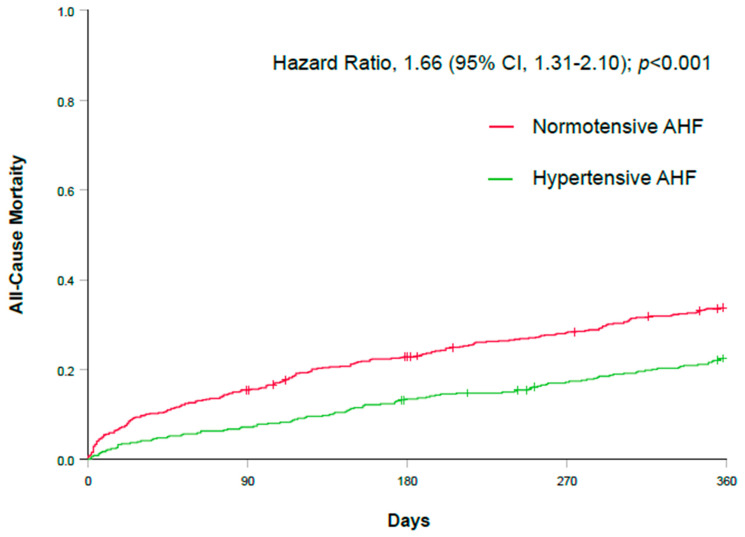
All-cause mortality during 360 days follow-up in normotensive and hypertensive AHF. AHF—acute heart failure; CI—confidence interval.

A total of 313 (28%) patients died in the 360-day follow-up period. At 360 days, mortality was higher in the normotensive AHF group as compared to the hypertensive AHF group (hazard ratio 1.66, 95% CI 1.31–2.10; *p* < 0.001; Figure 1).

**Figure 2:**
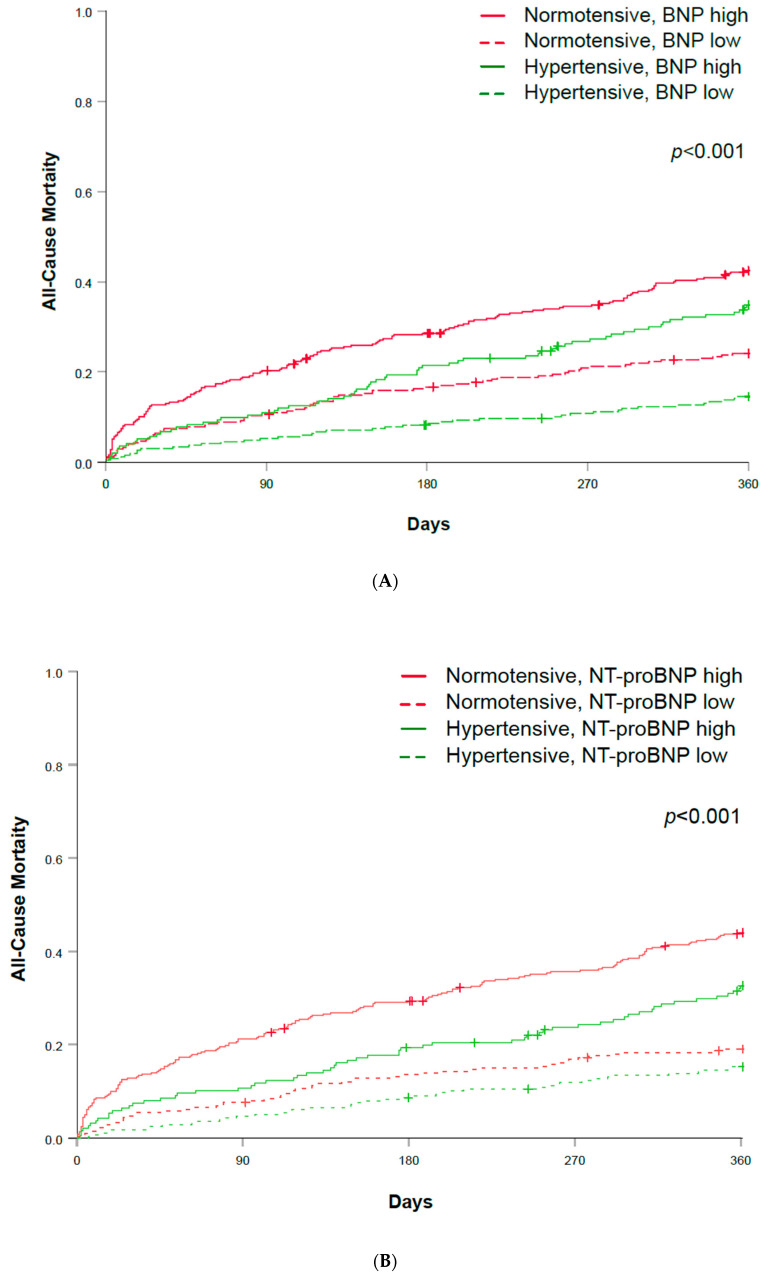
(**A**) All-cause mortality during 360 days of follow-up in normotensive and hypertensive AHF and according to BNP plasma concentration above or below the median. AHF—acute heart failure; BNP—B-type natriuretic peptide. (**B**) All-cause mortality during 360 days of follow-up in normotensive and hypertensive AHF and according to NT-proBNP plasma concentration above or below the median. AHF—acute heart failure; NT-proBNP—N-terminal prohormone of brain natriuretic peptide.

To better highlight the differential prognostic impact of admission blood pressure and hemodynamic stress, patients were divided into four groups: normotensive and hypertensive AHF patients with a low natriuretic peptide plasma concentration (<median, BNP in Figure 2A, NT-proBNP in Figure 2B) and normotensive and hypertensive AHF patients with a high natriuretic peptide plasma concentration (≥median). Quantifying hemodynamic stress by using the natriuretic peptide plasma concentration provided incremental prognostic information when classifying patients according to their normotensive versus hypertensive status at presentation. There was a significant difference in mortality rates between the four groups (Figure 2A,B, *p* < 0.001). Normotensive AHF patients with a high natriuretic peptide had the highest mortality.

**Figure 3:**
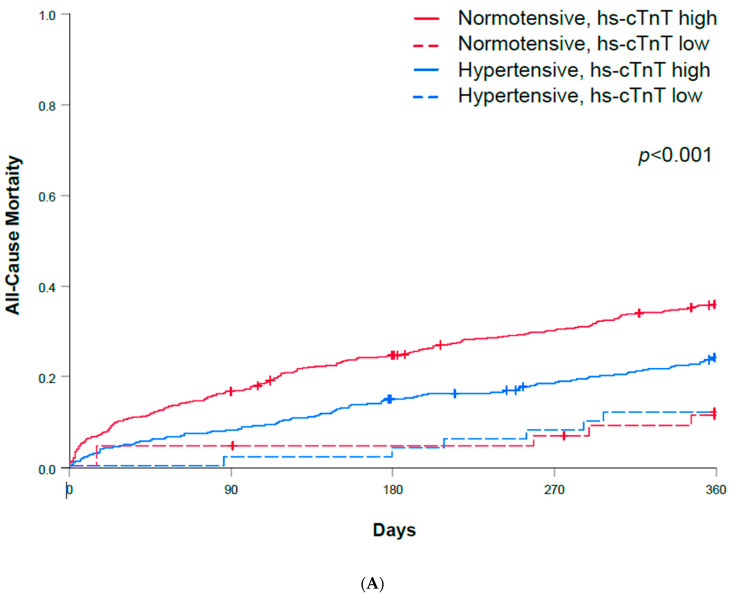

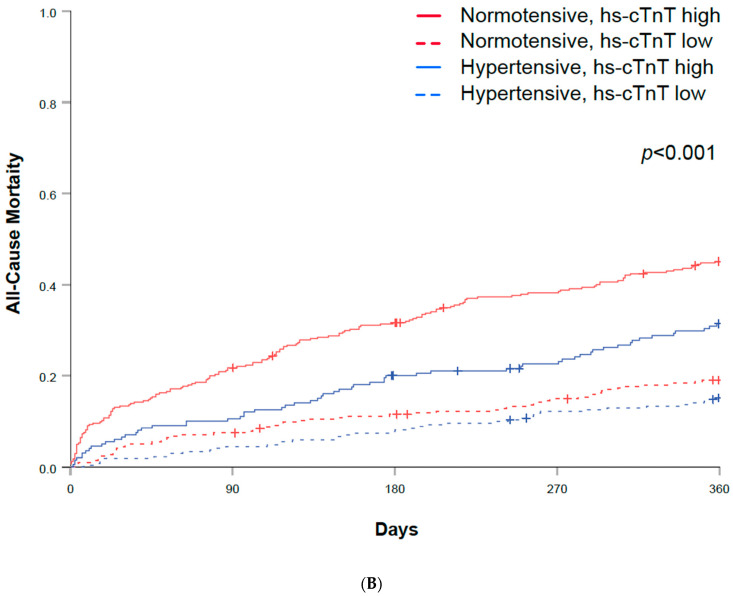
(**A**) All-cause mortality during 360 days of follow-up in hypertensive and normotensive AHF and according to hs-cTnT plasma concentration below or above the upper limit of normal. AHF—acute heart failure; hs-cTnT—high-sensitivity cardiac troponin T. (**B**) All-cause mortality during 360 days of follow-up in hypertensive and normotensive AHF and according to hs-cTnT plasma concentration below or above the median in the derivation cohort. AHF—acute heart failure; hs-cTnT—high-sensitivity cardiac troponin T.

Similarly, the prognosis was assessed in normotensive and hypertensive AHF patients with a normal hs-cTnT plasma concentration (<14 ng/L), as well as in normotensive and hypertensive AHF patients with elevated hs-cTnT (≥14 ng/L). There was a significant difference in the survival of the four groups (Figure 3A). Normotensive AHF patients with a high hs-cTnT plasma concentration had the highest mortality. Similar findings were demonstrated when using a hs-TnT cut-off at the median of 37 ng/L (Figure 3B).

Variables from the validated risk model to predict all-cause mortality were entered into multivariable Cox proportional survival analyses (Table 3A,B). Normotensive AHF was an independent predictor for all-cause mortality after a multivariable adjustment (*p* = 0.006). This was also true when testing in a model including normotensive AHF, hs-cTnT, NT-proBNP, and all variables from Table 1, which were significant in the univariable Cox proportional survival analyses (Table 3C). Notably, hs-cTnT was not an independent predictor in the multivariable model. Normotensive AHF remained an independent predictor of all-cause mortality when replacing NT-proBNP with BNP in the same model (Table 3D).

### 3.5. Validation Cohort

To externally validate the findings from the derivation cohort, hemodynamic cardiac stress and cardiomyocyte injury were quantified in the FINN-AKVA study. Among 324 patients eligible for this analysis, 132 (41%) had normotensive AHF.

Hemodynamic cardiac stress, as quantified by NT-proBNP, was significantly higher in normotensive AHF. Cardiomyocyte injury, as quantified by hs-cTnT, was significantly higher in normotensive AHF (Table S5).

At 360 days, all-cause mortality was higher in normotensive AHF as compared to hypertensive AHF (37% versus 19%, *p* < 0.001).

## 4. Discussion

This analysis within a large prospective diagnostic study was performed to address a significant gap in knowledge: the characterization of normotensive and hypertensive AHF phenotypes by quantifying hemodynamic cardiac stress and cardiomyocyte injury using natriuretic peptides and hs-cTnT as non-invasive quantitative tools. We report four major findings:

First, the prevalence of normotensive AHF was high, reaching nearly 60% in the derivation cohort and exceeding 40% in the validation cohort [19,20]. Second, de novo heart failure and history of hypertension were less common, and HFrEF was more frequent in normotensive AHF patients. These findings reflected the distinct pathophysiology and clinical presentation in the normotensive AHF phenotype [2,23,24,25]. Third, hemodynamic cardiac stress as well as the extent of cardiomyocyte injury were higher in normotensive AHF than in hypertensive AHF patients. The higher natriuretic peptide plasma concentrations might have been at least partly explained by the more common HFrEF as well as the significantly higher left ventricular end-diastolic diameter and the lower relative wall thickness, i.e., the higher wall stress, in patients with normotensive AHF. As wall stress seems to be the predominant trigger of natriuretic peptide release, the strong association between left ventricular end-diastolic diameter and end-diastolic wall stress results in higher hemodynamic cardiac stress in normotensive AHF [26,27].

In considering the relationship between normotension and cardiomyocyte injury, several interpretations are viable. Normotensive AHF patients may inherently sustain more cardiomyocyte damage. Alternatively, more pronounced myocardial injury could lead to a reduction in blood pressure, a pattern less severe than cardiogenic shock but present as a trend in this subgroup, especially in those with a reduced ejection fraction. Moreover, normotension might also represent a confounding factor, potentially a surrogate for more severe underlying disease states as indicated by the intensified use of medications such as spironolactone, beta blockers, and calcium antagonists. In addition, the higher hemodynamic cardiac stress, and the higher extent of cardiomyocyte injury in patients with normotensive AHF may reflect a more advanced stage of heart failure, evidenced by the higher prevalence of HFrEF and previous heart failure hospitalizations [28]. Such a progression likely entails a significant loss of contractile reserve and consequent hemodynamic impairment, potentially explaining the reduced tolerance to acute hemodynamic stress and the greater degree of cardiac necrosis observed in these patients [29]. These factors align with their higher mortality rates. These characteristics are associated with higher baseline (so-called “dry”) natriuretic peptide and cardiac troponin plasma concentrations [30,31]. Fourth, in normotensive AHF patients, adjusted and non-adjusted long-term survival was significantly lower. The higher proportion of de novo AHF patients, presumably with a higher contractile reserve as reflected by higher blood pressure, could explain the better survival in the hypertensive AHF group [23]. Furthermore, the hemodynamic profile of hypertensive AHF patients, characterized by a higher baseline blood pressure and stable cardiac output, may facilitate a timely and more extensive initiation of guideline-directed therapies such as ACE inhibitors, beta-blockers, and MRBs, which are often better tolerated in this group due to the reduced risk of symptomatic hypotension.

This study extended and corroborated findings from previous work on the characterization of AHF etiologies. While the high prevalence and some specific clinical features of normotensive vs. hypertensive AHF have been demonstrated in large registries, hemodynamic cardiac stress, cardiomyocyte injury, and their influence on prognosis have not been assessed in detail [7,23,25,32,33].

From a broader perspective, the findings of this study are of major importance due to the unmet clinical need for the sufficient characterization and understanding of AHF entities, which has left treating physicians for decades with very limited therapeutic options for this vulnerable patient population [34]. Our results, as well as everyday clinical practice, demonstrated that the normotensive AHF patients, often presenting with HFrEF, could represent a cohort with a more advanced stage of heart failure. These patients are characterized not only by the highest extent of hemodynamic cardiac stress and cardiomyocyte injury patterns, but also by the worst prognosis. They may less often be started on guideline-directed medical therapy, most likely due to difficulty in tolerating the medications. Furthermore, the predominance of HFrEF in normotensive AHF patients underscores a potentially greater impact from missed opportunities for guideline-directed treatment [28,35,36,37]. Notably, while our study provided an enhanced characterization of AHF entities, it did not directly address the nuanced therapeutic strategies for normotensive versus hypertensive AHF patients [38]. This study enrolled patients before sacubitril/valsartan was widely used. Accordingly, a confounding effect of sacubitril/valsartan on plasma BNP concentrations did not play a role for this analysis. Nevertheless, in current clinical practice, physicians should be aware of such an interaction when quantifying hemodynamic stress by using BNP in normotensive and hypertensive AHF patients treated with sacubitril/valsartan [31].

BASEL V had important methodological strengths, including its large sample size, highly representative patient population for acute dyspnea and AHF [39], and final diagnosis adjudicated by two independent cardiologists/internists according to current guidelines.

Several limitations of the study merit consideration. First, we cannot comment on the extent of hemodynamic cardiac stress and cardiomyocyte injury among patients with terminal renal failure, since such patients were excluded from our study. Second, the categorization of patients into the hypertensive AHF group was based solely on the first SBP measurement at ED presentation. This method did not account for the natural variability of SBP, including regression to the mean, which may occur without medical intervention. Moreover, any antihypertensive treatment received prior to ED presentation could have significantly influenced these initial BP readings. Furthermore, antihypertensive treatment by paramedics or family doctors prior to the ED presentation may have influenced blood pressure and, thereby, the allocation to groups. Third, the definition of hypertensive AHF was based on previous recommendations. However, the blood pressure cut-off is a matter of debate, and findings regarding hemodynamic cardiac stress and cardiomyocyte injury may vary according to the cut-offs chosen. Fourth, while the current analysis delineated some of the epidemiological differences between HFpEF and HFrEF within the AHF population, with a particular focus on the normotensive AHF cohort, where HFrEF is more prevalent, we recognize the complexity and the importance of these differences in informing therapeutic decisions. While our data provided initial insights, we acknowledge the need for further research to suggest treatment strategies.

## 5. Conclusions

This large multicenter international study demonstrated that normotensive AHF patients have a higher extent of hemodynamic cardiac stress and cardiomyocyte injury as compared to hypertensive AHF. Furthermore, normotensive AHF patients have worse survival, which may be explained not only by the phenotype associated with normal blood pressure per se, but also by the lower utilization of guidelines directing chronic heart failure therapy. This characterization might help raise awareness and individualize treatment strategies in these AHF phenotypes.

## Figures and Tables

**Table 1:** Patient characteristics.

	Overall	Normotensive AHF	Hypertensive AHF	*p*-Value
	n = 1152	n = 667	n = 485	
Age, median (IQR)	79 (71–85)	79 (72–85)	80 (70–85)	0.653
Female, n (%)	492 (43)	267 (40)	225 (46)	0.031
BMI, kg/m^2^	26 (23−30)	26 (23–30)	26 (24–30)	0.145
BSA *, m^2^	1.87 (1.71–2.03)	1.87 (1.70–2.01)	1.88 (1.71–2.05)	0.355
Medical history, n (%)				
Previous heart failure	558 (49)	365 (55)	193 (40)	<0.001
HFrEF **	356 (31)	258 (39)	98 (20)	<0.001
Previous hypertension ***	945 (82)	529 (80)	416 (86)	0.005
Coronary artery disease	607 (53)	374 (56)	233 (48)	0.008
COPD	174 (24)	163 (25)	117 (24)	0.880
Diabetes	348 (30)	195 (29)	153 (32)	0.399
Stroke	172 (16)	106 (17)	66 (14)	0.231
PAD	188 (18)	105 (17)	83 (18)	0.702
Medication at admission, n (%)				
ACE inhibitors	489 (43)	308 (47)	181 (38)	0.003
ARB	290 (26)	162 (25)	128 (27)	0.390
Beta-blockers	730 (64)	444 (67)	286 (60)	0.011
MRB	149 (13)	109 (16)	40 (8.3)	<0.001
Nitrates	186 (17)	115 (18)	71 (15)	0.229
Calcium channel blockers	255 (22)	122 (19)	133 (28)	<0.001
Digoxin	57 (5.2)	37 (5.9)	20 (4.3)	0.250
Antiarrhythmic drugs	133 (12)	92 (15)	41 (8.8)	0.004
Diuretics	798 (70)	497 (75)	301 (63)	<0.001
Clinical symptoms, n (%)				
Weight gain	415 (39)	270 (43)	145 (32)	<0.001
Orthopnea/PND	655 (59)	390 (61)	265 (57)	0.168
Chest pain	345 (30)	189 (29)	156 (32)	0.173
Coughing	581 (55)	341 (56)	240 (53)	0.247
Dyspnea duration (d), median (IQR)	7 (2–14)	7 (3–20)	5 (2–14)	0.002
Clinical signs, n (%)				
Elevated JVP	469 (45)	287 (47)	182 (41)	0.074
Rales	697 (64)	419 (66)	278 (60)	0.040
Peripheral oedema	721 (64)	421 (64)	300 (63)	0.625
Vital status				
Heart rate (bpm), median (IQR)	87 (71–105)	85 (71–105)	89 (72–105)	0.515
Oxygen saturation, median (IQR)	96 (93–98)	96 (93–98)	96 (93–98)	0.988
Laboratory values, median (IQR)				
Sodium, mmol/L	139 (136–141)	139 (136–141)	139 (137–141)	0.097
Hemoglobin, g/l	127 (114–141)	126 (112–141)	129 (116–141)	0.107
eGFR, mL/min/1.73 m^2^ ****	53 (36–74)	50 (35–70)	56 (37–78)	<0.001
BUN, mmol/L	10 (7–13)	10 (7–15)	8 (6–12)	<0.001
CK-MB, ng/mL	4.7 (3.4–6.9)	4.6 (3.4–6.9)	4.9 (3.4–6.8)	0.775
Echocardiography *****, median (IQR)				
LVEF, %	44 (30–55)	38 (25–55)	50 (36–60)	<0.001
LVEDD, mm	52 (46–59)	53 (47–61)	50 (45–57)	0.001
Septum, mm	10 (11–13)	11 (10–13)	12 (10–13)	0.020
Relative wall thickness	0.40 (0.32–0.48)	0.38 (0.30–0.47)	0.43 (0.35–0.51)	0.004
LVMI, g/m^2^	119 (94–149)	119 (95–151)	118 (94–145)	0.805
Diastolic dysfunction, n (%)	341 (30)	192 (29)	149 (31)	0.477

ACE inhibitors—angiotensin-converting enzyme inhibitors; ARB—angiotensin-receptor blocker; BUN—blood urea nitrogen; BMI—body mass index; BSA—body surface area; COPD—chronic obstructive pulmonary disease; CK-MB—creatine kinase-MB; eGFR—estimated glomerular filtration rate. IQR—interquartile range; JVP—jugular venous pressure; LVEDD—left ventricular end-diastolic diameter; LVEF—left ventricular ejection fraction; LVMI—left ventricular mass indexed for body surface area; MRB—mineralocorticoid receptor blockers; PAD—peripheral arterial disease; PND—paroxysmal nocturnal dyspnea; SBP—systolic blood pressure; TTE—transthoracic echocardiography. * Calculated by using the using the Mosteller formula. ** LVEF <40%; measurements of left ventricular internal diameters at end-diastole and end-systole were obtained from the parasternal long-axis view, and LVEF was calculated using the biplane method of disks (modified Simpson’s rule). *** Documented diagnosis of hypertension prior to the current episode of AHF, irrespective of antihypertensive treatment at admission. **** Calculated by using the CKD EPI formula. ***** Echocardiogram available in 882 patients; in 707 patients, the echocardiogram was performed during the index hospitalization (median time after admission 3 days (IQR 1–6).

**Table 2:** Cardiac stress and myocardial necrosis as quantified by BNP and hs-cTnT plasma concentrations.

	Overall	Normotensive AHF	Hypertensive AHF	*p*-Value
All patients				
BNP in pg/mL, median (IQR) (n = 1152)	974 (536–1712)	1105 (611–1956)	827 (448–1419)	<0.001
NT-proBNP in pg/mL, median (IQR)(n = 1105)	5161 (2338–9852)	5890 (2959–12,162)	4068 (1986–8118)	<0.001
hs-cTnT in ng/L, median (IQR) (n = 1152)	37 (22–67)	41 (24–71)	33 (19–59)	<0.001
HFrEF patients				
BNP in pg/mL, median (IQR) (n = 356)	1509 (914–2562)	1549 (1028–2637)	1157 (767–2116)	0.009
NT-proBNP in pg/mL, median (IQR) (n = 334)	7810 (4029–15,683)	8444 (4553–17,277)	5676 (3221–12,778)	0.008
hs-cTnT in ng/L, median (IQR) (n = 356)	42 (27–75)	43 (28–75)	40 (25–76)	0.515
HFmrEF patients				
BNP in pg/mL, median (IQR) (n = 153)	1011 (571–1573)	1111 (550–1740)	1013 (592–1455)	0.864
NT-proBNP in pg/mL, median (IQR)(n = 148)	5173 (2950–9059)	5173 (2914–9858)	5220 (2871–8826)	0.939
hs-cTnT in ng/L, median (IQR) (n = 363)	38 (19–71)	37 (23–65)	33 (19–59)	0.923
HFpEF patients				
BNP in pg/mL, median (IQR) (n = 363)	755 (367–1209)	730 (403–1173)	791 (351–1309)	0.481
NT-proBNP in pg/mL, median (IQR) (n = 357)	3612 (1672–7082)	4009 (1557–7654)	3996 (1735–6423)	0.624
hs-cTnT in ng/L, median (IQR) (n = 363)	32 (19–60)	37 (19–72)	30 (19–50)	0.018

NT-proBNP—N-terminal prohormone of brain natriuretic peptide; BNP—B-type natriuretic peptide; hs-cTnT—high-sensitivity cardiac troponin T; IQR—interquartile range; HFrEF—heart failure with reduced ejection fraction; HFmrEF—heart failure with mid-range ejection fraction; HFpEF—heart failure with preserved ejection fraction; SBP—systolic blood pressure.

**Table 3:** (**A**) Multivariable Cox proportional hazard model for mortality at 360 days in AHF patients using variables from a validated model (n = 1055). (**B**) Multivariable Cox proportional hazard model for mortality at 360 days in AHF patients using variables from a validated model (n = 1063). (**C**) Multivariable Cox proportional hazard model for 360-day mortality in AHF patients. This model incorporated normotensive AHF, NT-proBNP, and baseline predictors of all-cause mortality identified as significant in univariable models, utilizing all baseline characteristics detailed in Table 1 (n = 718). (**D**) Multivariable Cox proportional hazard model for 360-day mortality in AHF patients. This model incorporated normotensive AHF, BNP, and baseline predictors of all-cause mortality identified as significant in univariable models, utilizing all baseline characteristics detailed in Table 1 (n = 725).

(**A**)			
Variable	Hazard ratio	95% CI	*p*-Value
Normotensive AHF	1.393	1.090–1.781	0.008
Age (years)	1.038	1.024–1.052	<0.001
Beta-blockers at baseline	0.754	0.595–0.956	0.020
lg BUN (mmol/L)	4.342	2.378–7.928	<0.001
Hemoglobin (g/L)	1.001	0.995–1.007	0.675
lg NT-proBNP (pg/mL)	2.668	1.984–3.587	<0.001
(**B**)			
Variable	Hazard ratio	95% CI	*p*-Value
Normotensive AHF	1.412	1.106–1.804	0.006
Age (years)	1.043	1.029–1.057	<0.001
Beta-blockers at baseline	0.729	0.577–0.922	0.008
lg BUN (mmol/L)	6.306	3.561–11.165	<0.001
Hemoglobin (g/L)	1.001	0.995–1.007	0.731
lg BNP (pg/mL)	2.597	1.897–3.555	<0.001
(**C**)			
Variable	Hazard ratio	95% CI	*p*-Value
Normotensive AHF	1.368	1.003–1.865	0.048
lg NT-proBNP (pg/mL)	3.310	2.142–5.117	<0.001
hs-cTnT (ng/L)	0.803	0.505–1.278	0.355
Age	1.037	1.019–1.055	<0.001
Previous heart failure	1.143	0.842–1.552	0.391
Previous stroke	1.176	0.827–1.673	0.367
Elevated JVP	1.086	0.811–1.455	0.579
Oxygen saturation	0.964	0.941–0.988	0.003
Hemoglobin, g/l	1.003	0.996–1.011	0.427
Creatinine, µmol/L	1.003	1.001–1.005	0.003
LVEF	1.003	0.992–1.014	0.612
(**D**)			
Variable	Hazard ratio	95% CI	*p*-Value
Normotensive AHF	1.391	1.021–1.896	0.037
lg BNP (pg/mL)	3.312	2.095–5.235	<0.001
hs-cTnT (ng/L)	0.866	0.552–1.359	0.532
Age	1.043	1.026–1.061	<0.001
Previous heart failure	1.147	0.848–1.553	0.374
Previous stroke	1.228	0.866–1.742	0.248
Elevated JVP	1.036	0.776–1.385	0.808
Oxygen saturation	0.962	0.939–0.985	0.001
Hemoglobin, g/l	1.003	0.996–1.011	0.385
Creatinine, µmol/L	1.004	1.002–1.006	<0.001
LVEF	1.002	0.990–1.013	0.775

AHF—acute heart failure; BUN—blood urea nitrogen; CI—confidence interval; BNP—B-type natriuretic peptide; NT-proBNP—N-terminal prohormone of brain natriuretic peptide; hs-cTnT—high-sensitivity cardiac troponin T; LVEF—left ventricular ejection fraction; JVP—jugular venous pressure.

## Data Availability

The data presented in this study are available upon reasonable request from the corresponding author. The data are not publicly available.

## Supplementary Materials

The following supporting information can be downloaded at: https://www.mdpi.com/article/10.3390/biomedicines12051099/s1.

## Abbreviations

AHF	acute heart failure
BNP	B-type natriuretic peptide
CI	confidence interval
ED	emergency department
HFrEF	heart failure with reduced ejection fraction
hs-cTnT	high-sensitivity cardiac troponin T
NT-proBNP	N-terminal pro-B-type natriuretic peptide
SBP	systolic blood pressure
LVEF	left ventricular ejection fraction
