# Supplementary material for: Quantifying Hemodynamic Cardiac Stress and Cardiomyocyte Injury in Normotensive and Hypertensive Acute Heart Failure
Source: Biomedicines. 2024 May 16;12(5):1099. doi: 10.3390/biomedicines12051099 (PMC11118007; doi:10.3390/biomedicines12051099)
Supplement: Supplementary file 1 [file biomedicines-12-01099-s001.zip › biomedicines-2924773-supplementary.pdf]

Supplementary Material

Quantifying Hemodynamic Cardiac Stress and Cardiomyocyte Injury in Normotensive and Hypertensive Acute Heart Failure

Table 1S A Predictive values for BNP plasma concentrations above the median (n=848)

	**Odds ratio**	**95% CI**	***p*-value**
Normotensive AHF	0.976	0.718-1.328	0.878
Age	1.016	1.001-1.031	0.031
Female Sex	1.351	0.979-1.864	0.068
History of heart failure	1.160	0.850-1.582	0.349
History of hypertension	0.812	0.547-1.206	0.303
Creatinine, µmol/L	1.006	1.004-1.009	<0.001
LVEF, %	0.948	0.937-0.959	<0.001

AHF = acute heart failure; BNP= B-type natriuretic peptide; CI = confidence interval; LVEF= left ventricular ejection fraction on echocardiography; SBP= Systolic blood pressure

Table 1S B Predictive values for NT-proBNP plasma concentrations above the median (n=817)

	**Odds ratio**	**95% CI**	***p*-value**
Normotensive AHF	1.261	0.910-1.747	0.164
Age	1.041	1.024-1.058	<0.001
Female Sex	1.559	1.102-2.206	0.012
History of heart failure	1.139	0.819-1.585	0.439
History of hypertension	0.907	0.596-1.381	0.650
Creatinine, µmol/L	1.014	1.011-1.018	<0.001
LVEF, %	0.952	0.941-0.964	<0.001

AHF = acute heart failure; CI = confidence interval; LVEF= left ventricular ejection fraction on echocardiography; SBP= Systolic blood pressure

Table 2S A Predictive values for elevated (≥ 14 ng/L) hs-cTnT plasma concentrations (n=849)

	**Odds ratio**	**95% CI**	***p-value***
Normotensive AHF	1.021	0.594-1.754	0.941
Age	1.038	1.014-1.064	0.002
Female Sex	0.555	0.309-0.997	0.049
History of heart failure	0.927	0.525-1.638	0.795
History of hypertension	2.938	1.653-5.222	<0.001
Creatinine, µmol/L	1.030	1.018-1.042	<0.001
LVEF, %	0.969	0.951-0.988	0.001

AHF = acute heart failure; hs-cTnT = high-sensitivity cardiac Troponin T; CI = confidence interval; LVEF = left ventricular ejection fraction; SBP = systolic blood pressure

Table 2S B Predictive values for hs-cTnT plasma concentrations above the median (n=849)

	**Odds ratio**	**95% CI**	***p-value***
Normotensive AHF	1.169	0.866-1.578	0.309
Age	1.033	1.018-1.048	<0.001
Female Sex	0.625	0.457-0.853	0.003
History of heart failure	0.857	0.632-1.164	0.324
History of hypertension	1.118	0.763-1.637	0.568
Creatinine, µmol/L	1.008	1.006-1.011	<0.001
LVEF, %	0.977	0.967-0.988	<0.001

AHF = acute heart failure; hs-cTnT = high-sensitivity cardiac Troponin T; CI = confidence interval; LVEF = left ventricular ejection fraction; SBP = systolic blood pressure

Table 3S A Normotensive AHF prevalence according to Hemodynamic cardiac stress as quantified by BNP plasma concentrations

	**BNP low***	**BNP high**	***p*-value**
Normotensive AHF, n (%)	295 (51)	371 (65)	< 0.001

Table 3S B Normotensive AHF prevalence according to Hemodynamic cardiac stress as quantified by BNP plasma concentrations

	**NT-proBNP low***	**NT-proBNP high**	***p*-value**
Normotensive AHF, n (%)	276 (50)	364 (66)	< 0.001

Table 3S C Normotensive AHF prevalence according to cardiomyocyte injury as quantified by hs-cTnT plasma concentrations

	**hs-cTnT low****	**hs-cTnT high**	***p*-value**
Normotensive AHF, n (%)	47 (48)	620 (59)	< 0.001

Table 3S D Normotensive AHF prevalence according to cardiomyocyte injury as quantified by hs-cTnT plasma concentrations

	**hs-cTnT low***	**hs-cTnT high**	***p*-value**
Normotensive AHF, n (%)	307 (53)	360 (63)	< 0.001

* Below the median
** Below the upper limit of normal

BNP = B-type natriuretic peptide; hs-cTnT = high-sensitivity cardiac Troponin T

Table 4S A Medication regardless of LVEF

	**Overall**	**Normotensive AHF**	**Hypertensive AHF**	***p*-value**
**Medication at admission, n (%)**				
ACE inhibitors	489 (43)	308 (47)	181 (38)	0.003
ARB	290 (26)	162 (25)	128 (27)	0.390
Beta blockers	730 (64)	444 (67)	286 (60)	0.011
MRB	149 (13)	109 (16)	40 (8.3)	< 0.001
**Medication at discharge, n (%)**				
ACE inhibitors	653 (60)	373 (60)	280 (59)	0.913
ARB	285 (26)	159 (25)	126 (27)	0.570
Beta blockers	846 (77)	485 (77)	361 (77)	0.735
MRB	301 (29)	194 (32)	107 (24)	0.002

Table 4S B Medication of patients with HFrEF

	**Overall**	**Normotensive AHF**	**Hypertensive AHF**	***p*-value**
**Medication at admission, n (%)**				
ACE inhibitors	164 (47)	130 (51)	34 (35)	0.009
ARB	88 (25)	70 (28)	18 (19)	0.120
Beta blockers	228 (64)	180 (70)	48 (50)	< 0.001
MRB	72 (20)	61 (24)	11 (11)	0.010
**Medication at discharge, n (%)**				
ACE inhibitors	233 (69)	159 (66)	74 (77)	0.041
ARB	86 (26)	68 (28)	18 (19)	0.083
Beta blockers	282 (83)	199 (82)	83 (87)	0.311
MRB	155 (49)	110 (49)	45 (49)	0.941

Table 4S C Medication of patients with HFmrEF

	**Overall**	**Normotensive AHF**	**Hypertensive AHF**	***p*-value**
**Medication at admission, n (%)**				
ACE inhibitors	69 (46)	38 (48)	31 (43)	0.534
ARB	40 (27)	22 (28)	18 (25)	0.622
Beta blockers	96 (63)	50 (63)	46 (63)	0.972
MRB	22 (15)	17 (22)	5 (7)	0.010
**Medication at discharge, n (%)**				
ACE inhibitors	92 (62)	48 (62)	44 (62)	0.957
ARB	39 (26)	22 (28)	17 (24)	0.522
Beta blockers	119 (79)	61 (78)	58 (81)	0.722
MRB	44 (30)	27 (37)	17 (24)	0.101

Table 4S D Medication of patients with HFpEF

	**Overall**	**Normotensive AHF**	**Hypertensive AHF**	***p*-value**
**Medication at admission, n (%)**				
ACE inhibitors	131 (37)	71 (36)	60 (37)	0.917
ARB	97 (27)	36 (21)	61 (32)	0.021
Beta blockers	220 (61)	104 (61)	116 (61)	0.981
MRB	17 (4.7)	6 (3.5)	11 (5.7)	0.323
**Medication at discharge, n (%)**				
ACE inhibitors	185 (52)	86 (52)	99 (53)	0.920
ARB	99 (28)	37 (22)	62 (33)	0.021
Beta blockers	259 (73)	124 (75)	135 (72)	0.540
MRB	56 (16)	26 (16)	30 (16)	0.907

ACE inhibitor = angiotensin-converting enzyme inhibitor; ARB = angiotensin-receptor blocker; HFrEF = heart failure with reduced ejection fraction; HFmrEF = heart failure with mid-range ejection fraction; HFpEF = heart failure with preserved ejection fraction; MRB = mineralocorticoid receptor blockers; SBP = systolic blood pressure.

Table 5S Hemodynamic cardiac stress and cardiomyocyte injury as quantified by NT-proBNP and cTnT/cTnI plasma concentrations in the validation cohort

	**Overall**	**Normotensive AHF**	**Hypertensive AHF**	***p*-value**
NT-proBNP in pg/mL, median (IQR)	5,627 (2,605-11373)	7,225 (3,183-14,532)	4,833 (225-8,325)	< 0.001
cTnT in ng/L, median (IQR)	10 (10-70)	10 (10-90)	10 (10-60)	0.009
cTnI in ng/mL, median (IQR)	43 (14-217)	48 (19-294)	42 (12-179)	0.085

cTnI = cardiac Troponin I; cTnT= cardiac Troponin T; NT-proBNP = N-terminal pro-B-type natriuretic peptide; SBP = Systolic blood pressure

Supplemental figures

Figure 1S. Patient flow

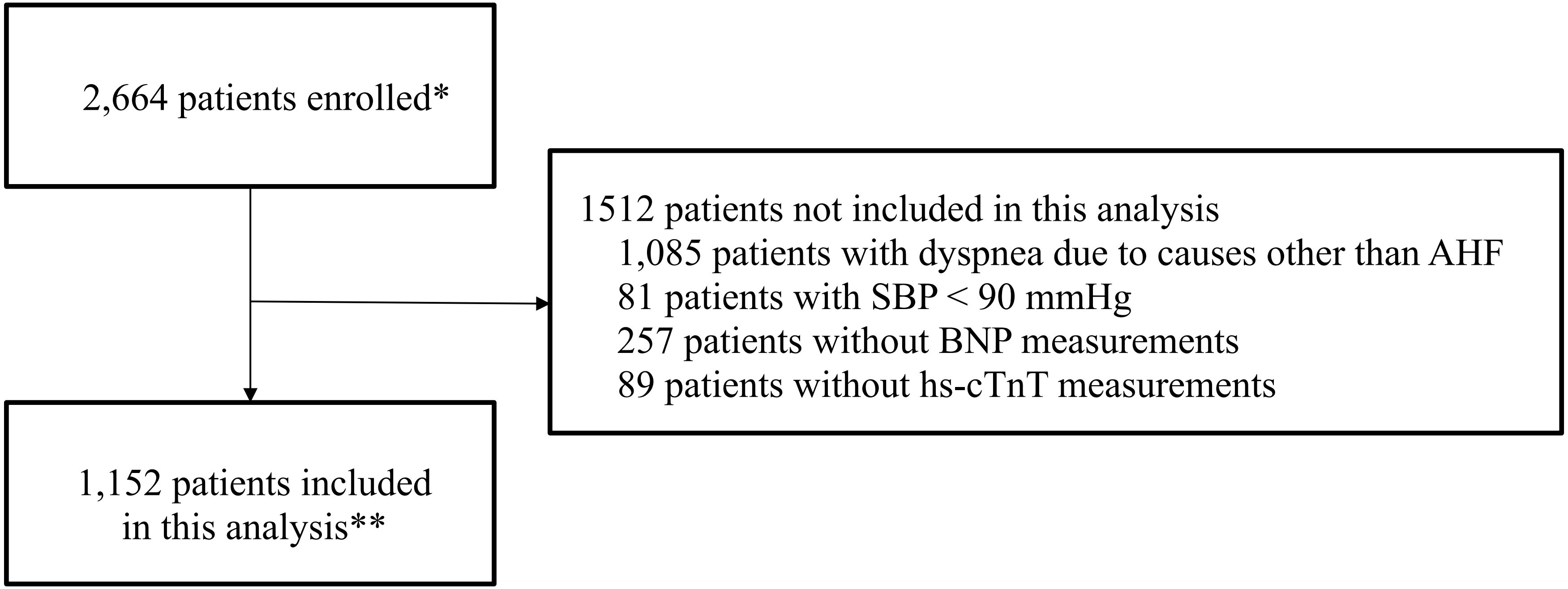

*Including 511 patients with a final diagnosis of AHF enrolled in an AHF therapy study until 2015 (ClinicalTrials.gov registry, Number NCT00512759).

**Including 309 patients with a final diagnosis of AHF enrolled in an AHF therapy study until August 2015 (ClinicalTrials.gov registry, Number NCT00512759).

AHF = acute heart failure; hs-cTnT = high-sensitivity cardiac Troponin T; BNP = B-type natriuretic peptide; SBP = systolic blood pressure
